# Knee Injury and Osteoarthritis Outcome Score: Validity and Reliability of an Indonesian Version

**DOI:** 10.31486/toj.20.0088

**Published:** 2021

**Authors:** Krisna Yuarno Phatama, Abdul Aziz, Muhammad Hilman Bimadi, I Gusti Ngurah Arga Aldrian Oktafandi, Felix Cendikiawan, Edi Mustamsir

**Affiliations:** Orthopaedic and Traumatology Department, Faculty of Medicine, Brawijaya University-Saiful Anwar General Hospital, Klojen, Malang, East Java, Indonesia

**Keywords:** *Activities of daily living*, *diagnostic self-evaluation*, *Indonesia*, *knee injuries*, *osteoarthritis–knee*, *pain measurement*, *quality of life*, *validation study*

## Abstract

**Background:** The Knee injury and Osteoarthritis Outcome Score (KOOS) is a useful diagnostic tool to assess knee ligament injury and osteoarthritis, but no validated Indonesian version of the KOOS was available.

**Methods:** We used the forward-backward translation protocol to develop the Indonesian version of the KOOS. The translated questionnaire was administered twice to 51 subjects diagnosed with a knee ligament injury and osteoarthritis. Validity of the questionnaire was assessed by analyzing the correlation between the score of each subscale and the overall score of the 36-Item Short Form Health Survey (SF-36) using the Pearson correlation coefficient. Reliability was measured by evaluating internal consistency (Cronbach α) and test-retest reliability (intraclass correlation coefficient).

**Results:** For construct validity, moderate Pearson correlation coefficients were found between the KOOS subscales and the SF-36. Cronbach α was 0.84 to 0.97 for all subscales, indicating adequate internal consistency. The test-retest reliability was excellent, with intraclass correlation coefficients ranging from 0.91 to 0.99 for all subscales. No significant differences were found in the KOOS subscale responses between the first administration of the questionnaire and the second administration within 21 days.

**Conclusion:** The Indonesian version of the KOOS was determined to be valid and reliable and is therefore an objective instrument for evaluating knee ligament injury and knee osteoarthritis in the Indonesian population.

## INTRODUCTION

Ligament injury frequently occurs during sports or as a result of trauma and is a structural, mechanical, and physiological change in the ligament that causes joint stability disruption.^1,2^ One of the most prevalent causes of knee pain, with an estimated prevalence of 20% in the adult population, ligament injury is associated with a substantially increased risk for the development of osteoarthritis in the patellofemoral and tibiofemoral joints.^1-3^

Tools for diagnosing knee ligament injury and osteoarthritis, such as the Lysholm Knee Scoring Scale and the Western Ontario and McMaster Universities Osteoarthritis Index (WOMAC), focus only on either the short-term or long-term consequences of knee ligament injury.^4,5^ Consequently, Roos and Lohmander developed an independent questionnaire as an extension of the WOMAC—the Knee injury and Osteoarthritis Outcome Score (KOOS)—to assess both short-term and long-term symptoms and function in patients with a knee ligament injury and osteoarthritis.^6^ The KOOS is a self-administered questionnaire for patients with anterior cruciate ligament injury, meniscus injury, or posttraumatic osteoarthritis and includes 42 items in 5 separately scored subscales related to symptoms, pain, activity of daily living, sport and recreation function, and quality of life (QOL).^6,7^

American English and Swedish versions of the KOOS were developed simultaneously, and KOOS translations are available in German, Danish, Russian, Italian, Spanish, French, Polish, Greek, Arabic, Portuguese, Persian, and Turkish.^8^ However, no validated knee ligament injury and osteoarthritis questionnaire was available in the Indonesian language. The objective of this study was to develop an Indonesian version of the KOOS and evaluate its validity and reliability.

## METHODS

### Study Design and Sample

The population in this cross-sectional study was 51 patients with a knee ligament injury and osteoarthritis from Saiful Anwar General Hospital and Persada Hospital, Malang, East Java, Indonesia. The inclusion criteria were presence of both a knee ligament injury and osteoarthritis, age of 18 to 70 years, and fluency in the Indonesian language. One orthopedist made the diagnosis of knee ligament injury and osteoarthritis in the 2 hospitals through history taking, physical examination, and radiology examination to ensure all patients had both a knee ligament injury and osteoarthritis. Exclusion criteria included age <18 years, age >70 years, and knee disorders other than knee ligament injury and osteoarthritis. Data were collected in February 2020. The hospital institutional review board reviewed and approved this study (ethical clearance number 400/056/K.3/302/2020).

### Development of the Indonesian Version of the KOOS

The first author (K.Y.P.) requested permission to develop an Indonesian version of the KOOS by sending an email to webmanager@koos.nu according to the 2012 KOOS guideline and was approved by Morten Pedersen on March 9, 2019. The forward-backward translation protocol was used during the translation process.

Two independent translators translated the American English questionnaire into Indonesian. One translator is an orthopedic expert, and the other is a professional translator. The 2 versions were then compared and discussed to correct any discrepancies. The resulting Indonesian translation was then translated back to English by one orthopedic expert and one professional translator. The resulting back-translation was assessed to confirm the similarity to the original American English version.

The Indonesian translation was given to 3 orthopedic experts for review, and an expert committee consisting of translators, health care workers, the authors, and academic methodology experts also assessed the translation for the similarity of each question to the original version and for ease of understanding. The committee's suggestions were used to design a prefinal version of the Indonesian KOOS.

### Preliminary Testing of the Indonesian Version of the KOOS and Finalization

The prefinal version of the Indonesian version of the KOOS was tested with 51 subjects with osteoarthritis and knee ligament injury to assess their understanding and interpretation of each item on the questionnaire. After making any necessary changes to the wording to ensure understanding, the committee finalized the Indonesian version of the KOOS.

### Research Procedure

The Indonesian version of the KOOS was used simultaneously with the Indonesian version of the 36-Item Short Form Health Survey (SF-36). The same 51 subjects who tested the prefinal version were asked to complete the Indonesian version of the KOOS and the SF-36 twice within an interval of 21 days.

The SF-36 is routinely used to assess health-related QOL,^9^ and the Indonesian version had been previously developed and studied.^10-12^ The 8 components assessed in the SF-36 are physical functioning, role physical, bodily pain, general health, vitality, social functioning, role emotional, and mental health. The first 4 components evaluate the physical health/physical components scale, and the other 4 assess the mental health/mental components scale. The SF-36 is widely used to assess several musculoskeletal problems, including knee osteoarthritis, as it evaluates general health aspects and is applicable to all age groups.^10,13^

### Statistical Analysis

The validity of an instrument can be determined by analyzing the instrument's correlation with other preexisting instruments that measure a similar outcome, a test that is also called construct validity. The construct validity of the Indonesian version of the KOOS was determined by analyzing the correlation between the score of each subscale and the overall score of the SF-36 using the Pearson correlation coefficient. *P* values <0.05 were deemed statistically significant. Pearson correlation coefficients of 0.1 to 0.3, 0.3 to 0.5, and >0.5 were considered weak, moderate, and strong, respectively.

The reliability test was divided into internal consistency and test-retest reliability. Internal consistency was measured by calculating the value of Cronbach α, and the test-retest reliability was evaluated by measuring the intraclass correlation coefficient (ICC) with a 95% CI.^14^ Cronbach α >0.70 was considered to denote adequate internal consistency. ICCs <0.50, of 0.50 to 0.75, of 0.75 to 0.90, and >0.90 were indicative of poor, moderate, good, and excellent reliability, respectively.^14,15^

Subscale scores on the Indonesian version of the KOOS from the first and second administrations within a 21-day interval were compared using the paired *t* test.

SPSS statistical software, version 25 (IBM Corp) for Microsoft Windows was used for all analyses.

## RESULTS

Of the 51 study subjects, 30 (58.8%) were males with a mean age of 36.4 ± 16.7 years, and 21 (41.2%) were females with a mean age of 50.2 ± 14.0 years.

The results of the validity test, presented by KOOS subscale, are shown in Table 1. The analysis showed a significant positive correlation between the score of each subscale and the overall score of the SF-36. All Pearson correlation coefficients were >0.30, indicating a moderate correlation as defined in the Methods section.

**Table 1.** Validity Test of the Indonesian Version of the Knee injury and Osteoarthritis Outcome Score (KOOS)

	36-Item Short Form Health Survey Overall Score	
KOOS Subscale	Pearson Correlation Coefficient	*P* Value
Symptoms	0.50	<0.001
Pain	0.51	<0.001
Activity of daily living	0.53	<0.001
Sport and recreation function	0.45	0.001
Quality of life	0.48	<0.001

Note: Pearson correlation coefficients between 0.1 and 0.3, between 0.3 and 0.5, and >0.5 indicate weak, moderate, and strong validity, respectively.

The results of the reliability test, presented by KOOS subscale, are shown in Table 2. All questionnaire subscales had Cronbach α values >0.70, denoting adequate internal consistency as defined in the Methods section. In the analysis of test-retest reliability, all ICCs were >0.90, indicative of excellent reliability as defined in the Methods section.

**Table 2.** Reliability Test of the Indonesian Version of the Knee injury and Osteoarthritis Outcome Score (KOOS)

KOOS Subscale	Internal Consistency Cronbach **α**	Test-Retest Reliability Intraclass Correlation Coefficient (CI)
Symptoms	0.84	0.97 (0.95-0.98)
Pain	0.94	0.91 (0.87-0.94)
Activity of daily living	0.97	0.99 (0.98-0.99)
Sport and recreation function	0.96	0.98 (0.96-0.99)
Quality of life	0.86	0.99 (0.98-0.99)

Notes: Cronbach α >0.70 denotes adequate internal consistency. Intraclass correlation coefficients <0.50, between 0.50 and 0.75, between 0.75 and 0.90, and >0.90 indicate poor, moderate, good, and excellent reliability, respectively.

The Figure shows mean scores for each KOOS subscale at the 2 administrations of the questionnaire. The results of the paired *t* test showed no significant differences in the mean scores between the first and the second administrations for each subscale.

**Figure.**
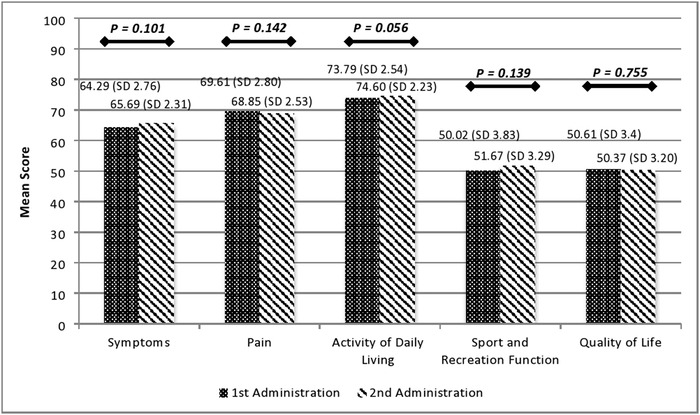
**Comparison of mean subscale scores between the first (top scores) and the second (bottom scores) administrations of the Indonesian version of the Knee injury and Osteoarthritis Outcome Score.**

## DISCUSSION

Our analysis of the construct validity of an Indonesian version of the KOOS demonstrated a significant positive correlation between the score of each KOOS subscale and the overall score of the SF-36. All *P* values were <0.001, and all Pearson correlation coefficients were >0.30, indicating a moderate correlation. Hence, the results confirmed the validity of the questionnaire. Studies by Roos et al,^7^ Cheung et al,^16^ and Gonçalves et al,^17^ also showed a moderate correlation between the KOOS and the SF-36. This moderate correlation is caused by the generic nature of the SF-36 that emphasizes the assessment of overall QOL (including mental health) and is therefore less responsive than the KOOS for assessing knee-specific symptoms, function, and QOL.^16,18,19^

Both the internal consistency and the test-retest reliability results confirmed the reliability of the Indonesian version of the KOOS. We found that all the KOOS subscales on the Indonesian version have adequate internal consistency, with all Cronbach α values >0.70 (range, 0.84-0.97). These values are comparable to those for the Finnish version^20^ (Cronbach α range, 0.79-0.96) and the Malaysian version^21^ (Cronbach α range, 0.78-0.95). Our test-retest reliability analysis showed that the Indonesian version of the KOOS has excellent reliability, with all ICC values >0.90 (range, 0.91-0.99). Our results are similar to those for the Italian version^22^ (ICC range, 0.85-0.95) and are higher than the results for the Finnish version^20^ (ICC range, 0.73-0.86).

Finally, the comparison of mean subscale scores for the Indonesian version of the KOOS that were obtained at 2 timepoints within a 21-day interval showed no significant differences (*P*>0.05).

To our knowledge, this study is the first validation of an Indonesian language instrument that evaluates knee ligament injury and osteoarthritis. However, our study has limitations. We only used the SF-36 to measure the construct validity of the Indonesian version of the KOOS. However, no other Indonesian language instruments were available with similar constructs to the KOOS. Thus, further study in adapting and validating other instruments against the Indonesian version is necessary. In addition, we did not analyze the responsiveness of the instrument, so the ability of the instrument to detect clinical changes over time is unknown.

## CONCLUSION

The Indonesian version of the KOOS exhibited adequate internal consistency, excellent test-retest reliability, and moderate construct validity and is therefore an objective tool for evaluating knee ligament injury and osteoarthritis in the Indonesian population. However, additional adaptation and validation studies of similar instruments are needed.

## Notes

This article meets the Accreditation Council for Graduate Medical Education and the American Board of Medical Specialties Maintenance of Certification competencies for Patient Care, Medical Knowledge, and Practice-Based Learning and Improvement.
